# Biological Efficacy Evaluation of a Non-Cross-Linked Hyaluronic Acid Dermal Filler for Biomedical Application in Inflammatory Scalp Conditions

**DOI:** 10.3390/life12010002

**Published:** 2021-12-21

**Authors:** Sabrina Sommatis, Maria Chiara Capillo, Elsa Liga, Cristina Maccario, Raffaele Rauso, Martha Herrera, Nicola Zerbinati, Roberto Mocchi

**Affiliations:** 1 UB-CARE S.r.l.-Spin-Off, University of Pavia, 27100 Pavia, Italy; sabrina.sommatis@ub-careitaly.it (S.S.); mariachiara.capillo@ub-careitaly.it (M.C.C.); quality@ub-careitaly.it (E.L.); 2 Maxillofacial Surgery Unit, Department of Medicine and Surgery, University of Campania “Luigi Vanvitelli”, 81100 Naples, Italy; cristina.maccario@ub-careitaly.it (C.M.); raffaele.rauso@unicampania.it (R.R.); 3 Centro Avanzado de Dermatologia y Laser, San Pedro Sula 21104, Honduras; martha@dermahn.com; 4 Department of Medicine and Surgery, University of Insubria, 21100 Varese, Italy

**Keywords:** keratinocytes, inflammation, antimicrobial, biofilm, Reconstructed Human Epidermis, scalp disorders, mesotherapy, hyaluronic acid, microbial and yeast infections, interleukin (IL)-8

## Abstract

(1) Background: The dysbiosis of some cutaneous commensal microorganisms is the trigger factor for the activation of the inflammatory cascade by keratinocytes in many skin disorders. Mesotherapy is an innovative technique for many scalp disorders, with the function of restoring the physiology of the skin. (2) Methods: the antimicrobial, antibiofilm and anti-inflammatory activity of the non-cross-linked HA formulation (Hydro Deluxe, Matex Lab S.p.a., Brindisi, Italy) was investigated against the most common microorganisms of the scalp (*Staphyloccoccus epidermis, Staphyloccoccus aureus, Cutibacterium acnes* and *Malassezia furfur).* Anti-inflammatory activity was evaluated on an internal 3D model of Reconstructed Human Epidermis (RHE) inserts infected with the strains as pro-inflammatory stimulus. (3) Results and Conclusions: the data collected showed a good antimicrobial and antibiofilm activity against all selected strains. The HA-based formulation did not show cytotoxicity on RHE, either alone or in presence of the infectious stimulus. The analysis of the expression of Interleukin (IL)-8 levels showed an excellent ability to reduce this pro-inflammatory marker. Overall, the efficacy assessment of the formulation supported its potential effectiveness in mesotherapy for the treatment of scalp disorders.

## 1. Introduction

Epidermis is the outermost of the three layers that make up the skin. Its main function is as a barrier against external injury and environmental pathogen infection. The epidermis architecture includes the *stratum basale* (the deepest portion of the epidermis), *stratum spinosum*, *stratum granulosum*, *stratum lucidum*, and *stratum corneum* (the most superficial portion of the epidermis). *Stratum basale*, also known as stratum germinativum, presents from cuboidal to columnar cells, mitotically active stem cells that are constantly producing keratinocytes. *Stratum spinosum* contains irregular, polyhedral cells with cytoplasmic processes and dendritic cells. *Stratum granulosum* contains diamond-shaped cells with keratohyalin granules (rich of keratin precursors) and lamellar granules (rich of glycolipids). *Stratum lucidum* is a thin clear layer consisting of eleidin (the transformation product of keratohyalin) [1]. *Stratum corneum* is the uppermost layer, rich in keratin and horny scales made up of dead keratinocytes known as anucleate squamous cells. It performs protective and adaptive physiological functions, including mechanical shear, water flux and hydration regulation, microbial proliferation and invasion regulation (secreting defensins), and initiation of inflammation through cytokine activation and dendritic cell activity [2]. Keratinocytes are the predominant cell type of epidermis and they originate in the basal layer, providing the structural and physical integrity of the skin and protecting the body against foreign antigens and microorganisms. They also play an active role in the generation and expression of protective immune responses and immunopathological reactions. The constitutive production of immune and inflammatory mediators by unperturbed keratinocytes is very contained, but can be enhanced by various trigger factors such as ultraviolet radiation, irritants, microorganisms, microbial products, tumor promoters, and trauma/wound repair [3].

Among immunomodulating cytokines, Interleukin (IL)-8, a potent chemoattractant for neutrophils, was reported to be induced constitutively from keratinocytes and, in upregulated ways, in many inflammatory skin disorders, such as psoriasis, dandruff, seborrheic and atopic dermatitis, and cicatricial alopecia (androgenetic alopecia (AGA), alopecia areata (AA) and non-cicatricial (telogen effluvium) types) [4,5,6,7].

Nowadays, it is widely recognized that microbial species such as *Cutibacterium acnes (C. acnes), Staphylococcus aureus (S. aureus), Staphylococcus epidermidis (S. epidermidis)* and the yeast *Malassezia furfur (M. furfur)* are commensal species of the skin and play a key role in innate skin immune system activity. However, the same members of the microbiological skin flora are also involved in the pathogenesis of cutaneous inflammatory disorders, especially under altered microenvironmental conditions related to surface water content, skin surface pH, or increased sebaceous activity [8,9]. In particular, increased Sebum Excretion Rate (SER) promotes hypercolonization of the microbial flora playing a key role in the onset of scalp disorders such as seborrheic dermatitis and dandruff. Sebum is produced by sebaceous glands (SGs), secretary organs located near hair follicles, and it is a complex mixture of fatty acids (57%), wax esters (26%), squalene (12%), and cholesterol (4.5%) that forms a protective hydrolipidic barrier which has the function of waterproofing the hair shaft and protecting the skin from external environmental agents [10]. This baseline production of sebum is called physiological seborrhea and plays a beneficial role for the skin. On the other hand, the hyperactivity of the SGs and the related overproduction of sebum are associated with a pathological form of seborrhea. Therefore, sebum provides an optimal nutrient source for the skin microflora that could break down triglycerides and esters into diglycerides, monoglycerides and free fatty acids, promoting its adherence and providing sources of nutrients for yeast such as *M. furfur*, which does not produce its own lipids and may benefit from the sebum lipids. Furthermore, some strains, such as *C. acnes* and *S. epidermidis*, are able to establish macrocolonies or biofilm communities between squamous cells of the outer stratified epithelium—or could penetrate deep in the SG epithelial surfaces, making them more resistant against innate immune cells or antimicrobial agents [11]. Altogether, this creates irritation and inflammation on the scalp, leading to intense itchiness and obstruction of natural hair growth and causing hair loss (alopecia) with different degrees of severity. Treatments depend on the location, incidence and age of the person, and include: local treatment with products containing coal tar, ketoconazole, salicylic acid, selenium sulfide, or zinc pyrithione; topical applications of creams or ointments containing hydrocortisone, fluocinolone, clobetasol, or desonide; and antifungal medication. For the most severe forms of alopecia, innovative practices such as mesotherapy, microneedling, platelet-rich plasma (PRP), low-level light therapy, and stem-cells have been proposed [12].

Mesotherapy is a minimally invasive technique consisting of local intradermal therapy (LIT) by multiple injections of active substances. Various compounds, including nutrients, hormones, vitamins, enzymes and other biocompatible and absorbable reagents that can be injected intradermally or subcutaneously to reach the target tissues and promoting restoration of healthy skin, are used in this technique [13]. There is evidence regarding the clinical efficacy of mesotherapy in the treatment of skin aging, local pain and fat contouring, but evidence-based studies regarding the efficacy of mesotherapy in different types of alopecia are few in number and controversial. Recently, mesotherapy has increasingly been used in the treatment of cicatricial and non-cicatricial alopecia types [14,15,16]. However, optimal protocols to achieve satisfying results are dependent on the quality of medication formulas and the mode of their application. Non-cross-linked hyaluronic acid (HA) represents an ideal bio-renewable polymer for this purpose, because it is an anionic, non-sulfated glycosaminoglycan distributed throughout the human body but also widely present in different species. Among its biological functions, hyaluronic acid participates in wound healing, modulation of inflammatory mediators, interaction with proteoglycans of the extracellular matrix and scavenging of free radicals. Local application of HA-based compounds has also been reported to exert antimicrobial activity against various infectious agents in a dose-dependent manner; in association to the ability to inhibit the growth of different planktonic cells, HA’s ability to reduce bacterial adhesion and biofilm aggregates has been recognized, a feature that makes it suitable for the control of microbial proliferation in inflammatory sites [17]. High biocompatibility, antiadhesive properties, and non-immunological safety profiles make HA-based formulations a possible non-antibiotic option to reduce the impact of infections related to microbial over-proliferation or biofilm formation in various inflammatory disorders. In this work, the antimicrobial activity of a non-cross-linked HA based formulation (Hydro Deluxe, Matex Lab S.p.a, Brindisi, Italy) was investigated against the microbial strains most involved in skin disorders of the scalp. Its efficacy was also screened against the preformed biofilms of the same strains. In order to better investigate the anti-inflammatory activity of the formulation in a scalp inflammatory condition, a Reconstructed Human Epidermis (RHE) model stimulated with the four selected strains was developed and screened for IL-8 production as a keratinocytes-related pro-inflammatory biomarker.

## 2. Materials and Methods

### 2.1. Sample Collection

The study was performed on a non-cross-linked hyaluronic acid (HA)-based formulation (Hydro Deluxe) containing 18 mg/mL HA, 0.01% Calcium Hydroxyapatite (CaHA), Glycine and L-Proline provided by Matex Lab S.p.a (Brindisi, Italy).

### 2.2. Microbial Strain and Reconstructed Human Epidermis 3D Model

Four different microbial strains were selected for the in vitro investigation objective of our study, each cultured in specific media and growth conditions. *C. acnes* ATCC 11827 (Microbiologics, MN, USA) was cultured in Brain Heart Infusion (BHI, VWR Chemicals, Milano, Italy) broth for the liquid growth of the strain and in Sheep Blood Agar (Biolife Italiana, Milan, Italy) for the growth of colonies in plate. The bacteria were cultured in an anaerobic atmosphere using BBL GasPak systems (Becton Dickinson Microbiology Systems, Cockeysville, MD, USA) at 35 ± 2 °C. *S. aureus* ATCC 6538 (Mecconti S.A.R.L. Sp.z.o.o., Warszawa, Poland) was cultured in Tryptic Soy Broth (TSB, Acumedia LAB Neogen, St. Cloud, MN, USA) for the liquid and Tryptic Soy Agar (TSA, Condalab, Madrid, Spain) for the growth of colonies in plate 35 ± 2 °C. *S. epidermidis* ATCC 12228 (Microbiologics, St. Cloud, MN, USA) was cultured in TSB for the liquid growth and TSA for the growth in plate at 35 ± 2 °C. The yeast *M. furfur* ATCC 14521 (Mecconti S.A.R.L. Sp.z.o.o., Warszawa, Poland) was cultured in Sabouraud Dextrose Broth (SDB, Condalab, Madrid, Spain) added with 0.5% Tween 80 (Sigma Aldrich, St. Louis, MO, USA) for the liquid growth and Sabouraud Dextrose Agar (SDA, Condalab, Madrid, Spain) with 0.5% Tween 80 added for the growth in plate at 28 ± 2 °C.

Reconstructed Human Epidermis (RHE) 3D model (Episkin^®^ Laboratories, Lyon, France) was supplied by SkinEthic™ laboratories; it is a reconstructed tissue made from normal human keratinocytes grown for 17 days that consists in a fully differentiated epidermis layered on a 0.5 cm^2^ inert polycarbonate filter.

### 2.3. In Vitro Evaluation of the Anti-Microbial Activity

In order to verify the antimicrobial efficacy of the hydrogel against the reference strains selected, the Minimum Inhibitory Concentration (MIC) protocol was performed using the 96-well microtiter plates method [18]. Serial dilutions of the non-cross-linked HA hydrogel were prepared according to the following ratio between sample and broth (specific for the better growth conditions of each strain) for microbial growth: formulation in toto and subsequent dilutions 1:2 (range tested from 100% to 0.78%) with respect to the control (in absence of tested formulation). For each dilution, 180 µL were dispensed into the respective well and 20 µL of the calibrated inoculum at the concentration of 10^7^ CFU/mL were added to each well. In parallel, an equal number of wells were prepared with the same dilutions reported above without adding microbial strains; these wells were used as background for subsequent optical density (OD) correction readings. The plates were then incubated at optimal growth conditions for the selected strains, as follows: 24 h at 35 ± 2 °C in aerobiosis for *S. aureus* ATCC 6538 and *S. epidermidis* ATCC 12228; 24 h at 35 ± 2 °C in anaerobiosis GasPak system for *C. acnes* ATCC 11827; 24 h at 28 ± 2 °C for *M. furfur* ATCC 14521. The same protocol was followed for all reference strains. After 24 h of incubation, the spectrophotometric reading was performed at a wavelength of 595 nm using a microplate reader (MultiskanTM Go, Thermo Scientific, Massachusetts, USA). The value of the background (hydrogel dilutions without microbial strains inoculated) was subtracted from each reading. Thus, the delta of absorbance was calculated, which measures the OD represented only by the bacterial load present in the well.

### 2.4. Biofilm Metabolic Activity Assay

The metabolic activity of preformed biofilm was determined by 3-[4,5-dimethylthiazol-2-yl]-2,5 diphenyl tetrazolium bromide (MTT) assay with same modifications [19]. For the cultivation of biofilm, 200 µL/w of a broth culture (in BHI for *C. acnes* ATCC 11827; TSB for *S. aureus* ATCC 6538 and *S. epidermidis* ATCC 12228; SDB + Tween 80 for *M. furfur* ATCC 14521) at 10^8^ CFU/mL density were seeded in a flat-bottomed polystyrene 96-well plate (VWR, Milano, Italy) and incubated at optimal growth conditions for each strain under static conditions [20]. The preformed biofilm of each strain was treated for 24 h as follows: 200 µL/w of fresh Broth Media for negative control; 200 µL/w of the non-cross-linked HA hydrogel in toto; 200 µL/w of a compound scientifically recognized as bioactive on the preformed biofilm of the selected strains: trans-resveratrol, 3,5,4′-trihydroxy-trans-stilbene (Resveratrol, Millipore, Burlington, MA, USA) at 394 µg/mL concentration for the treatment of the *C. acnes* ATCC 11827 preformed biofilm; 2-Aminoimidazole (Sigma Aldrich, St. Louis, MO, USA) 50 µM for the treatment of *S. aureus* ATCC 6538 biofilm; Cynnamaldeide 1.2 mg/mL (Sigma Aldrich, St. Louis, MO, USA) for the eradication of the *S. epidermidis* ATCC 12228 preformed biofilm; Ketaconazole (Sigma Aldrich, St. Louis, MO, USA) at 32 µg/mL for the treatment of *M. furfur* ATCC 14521 biofilm. Each plate was incubated for an additional 24 h at optimal growth conditions [21,22,23,24]. After the incubation period, the well contents were discarded gently using a single-channel micropipette and each well was washed twice with 200 µL/w DPBS and air-dried at 60 °C for 60 min to heat-fix the attached biofilm at the bottom of the well. The MTT solution (5 mg/mL in DPBS) was dispensed (100 µL/w) and the plate was incubated for 3 h at 37 °C. The insoluble purple formazan crystals were dissolved in 100 µL/w of Dimethyl Sulfoxide (DMSO, Honeywell, Carolina del Nord, USA) and the optical density (OD) was measured at 570 nm using a microplate rider. The metabolic activity related to total biofilm mass in each tested condition was calculated as:

Biofilm Metabolic Activity (%) = [OD_570 nm_ test product/OD_570 nm_ negative control] × 100 (1)

### 2.5. In Vitro Evaluation of the Cytotoxicity on RHE 3D Model Stimulated with Microbial Strains

To verify the efficacy of our RHE model stimulated with different microbial strains, a preliminary cytotoxicity evaluation of the treatment conditions was performed. Before using the microbial strains as pro-inflammatory stimuli on keratinocytes 3D model, the strains were cultured in their respective culture broths for 24 h under specific culture conditions. The log-phase bacterial cultures were harvested (10^8^ CFU/mL), washed (×3) with Dulbecco Phosphate Buffer Saline (DPBS, Sigma Aldrich, St. Louis, MO, USA) and incubated at 80 °C for 30 min to induce a thermal shock. Finally, heat-killed bacteria were stored at 4 °C until use [25]. Thermal inactivation is a recognized inactivation method to preserve bacterial surface proteins able to initiate the inflammatory process by preventing the over-proliferation of bacterial cells [26].

After arrival, the RHE inserts were placed in a growth medium (Episkin^®^ Laboratories, Lyon, France) under sterile conditions and incubated at 37 °C, 5% CO_2_, overnight. After the equilibration period, 20 μL of the tested formulation were applied in toto on the surface of the epithelium insert for 24 h. This contact can mediate the penetration of various compounds to the deeper layers of the epidermis, similar to physiological conditions. In parallel, the viability of the inserts was evaluated after co-treatment with 20 μL of the hydrogel and 100 μL of a calibrated inoculum (10^8^ CFU/mL) of each selected heat-killed strain (*C. acnes* ATCC 11827, *S. aureus* ATCC 6538, *S. epidermidis* ATCC 12228 and *M. furfur* ATCC 14521) representing the inflammatory stimulus. Furthermore, control inserts (Ctrl) were treated with DPBS and with the positive control, consisting of 100 μL of a calibrated inoculum (10^8^ CFU/mL) of each microbial strain (*C. acnes* ATCC 11827, *S. aureus* ATCC 6538, *S. epidermidis* ATCC 12228 and *M. furfur* ATCC 14521).

Each condition, including positive and negative controls, was evaluated in duplicate. At the end of the treatment (24 h), RHE inserts were rinsed with DPBS (×25) with a continuous flow maintained at 5–8 cm distance, avoiding splashing and contaminations. The bottom of each insert was hit on sterile gauze and transferred to a 24-well plate filled with 300 µL of 3-[4,5-dimethylthiazol-2-yl]-2,5 diphenyl tetrazolium bromide (MTT, Sigma Aldrich, St. Louis, MO, USA) solution (1 mg/mL), then incubated for 3 h at 37° C, 5% CO_2_. After extraction with isopropanol, OD of the samples was quantified by spectrophotometry at 570 nm wavelength using a microplate reader.

The viability of the RHE in each condition was calculated as a ratio of the corrected optical densities of the sample over the negative control (untreated sample).

Cell viability (%) = [OD_570 nm_ test product/OD_570 nm_ negative control] × 100 (2)

Cell viability values ≤ 50% are indexes of irritation according to the reference standard method of skin irritation (DB-ALM Protocol n° 135: SkinEthic™ Skin Irritation Test) [27].

### 2.6. In Vitro Evaluation of the Anti-Inflammatory Activity on RHE Stimulated

After verifying the cell viability of the infected RHE models, the treatment protocol was performed again in order to screen the RHE culture medium for Interleukin (IL)-8 pro-inflammatory cytokine by an ELISA kit (Thermo Scientific, Waltham, MA, USA) following the manufacturer’s instructions. The absorbance was then measured at 450 nm using a microplate reader and the IL-8 quantification was obtained, plotting the mean absorbance of each sample with a 5PL standard curve (15.6–1000 pg/mL).

### 2.7. Statistical Analysis

All data are presented as the mean ± standard deviation (SD) of two independent experiments. All graphs and statistical analyses were performed using GraphPad Prism software version 9.0 (GraphPad Software, San Diego, CA, USA). Analysis of variance and significant differences among means were tested by one-way ANOVA, followed by Bonferroni’s multiple comparisons post-test where appropriate. Differences were considered significant when *p* ≤ 0.05.

## 3. Results

### 3.1. Antimicrobial Activity Ogainst the Selected Strains

**Figure 1:**
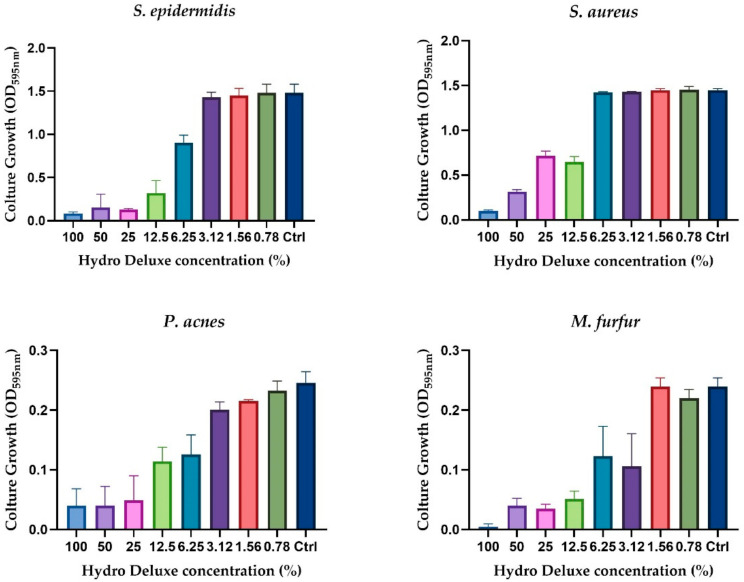
Graphical representation of the antimicrobial activity of the non-cross-linked HA hydrogel (Hydro Deluxe) against the selected strains (*S. aureus* ATCC 6538, *S. epidermidis* ATCC 12228, *C. acnes* ATCC 11827 and *M. furfur* ATCC 14521). Results are shown as culture growth (OD_595nm_) of the selected bacteria and yeast compared with the control (Ctrl, medium inoculated with 10^6^ CFU/mL of the strains) after 24 h of incubation with 8 serial dilutions of the formulation (range tested 100–0.78%).

The evaluation of the antimicrobial activity showed that the non-cross-linked HA based formulation was able to inhibit the growth of the selected strains (*S. epidermis, S. aureus, C. acnes* and *M. furfur*) in a concentration-dependent manner. Figure 1 shows the graphical representation of the microbial growth after 24 h of direct contact with serial dilution (ratio 1:2) of the product, starting from the 100% (in toto) to 0.78%. Broth culture media inoculated with each of the selected strains were used as control (Ctrl) for each experiment performed (*n* = 2; *r* = 3).

### 3.2. Antibiofilm Evaluation

**Figure 2:**
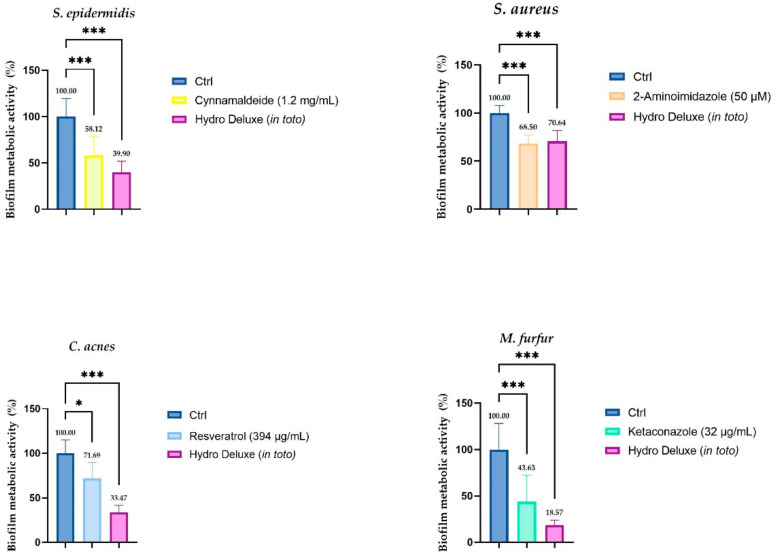
Biofilm metabolic activity (%) of a preformed biofilm after 24 h of treatment with the non-cross-linked HA hydrogel (Hydro Deluxe) 100% (in toto). For each strain, in association with the effect of the HA-based formulation, the efficacy of a compound with recognized antibiofilm activity is evaluated. The baseline condition (Ctrl) is represented by the preformed biofilm treated with fresh medium. The positive controls selected for each strain are: Cynnamaldeide (1.2 mg/mL) for *S. epidermidis,* 2-Aminoimidazole (50 µM) for *S. aureus*, Resveratrol (394 µg/mL) for *C. acnes* and Ketaconazole (32 µg/mL) for *M. furfur*. Values of * *p* ≤ 0.05 and *** *p* ≤ 0.001 were considered statistically significant compared with respective controls by one-way ANOVA statistical analysis, followed by Bonferroni’s multiple comparisons as post-test.

The results obtained from the incubation of the product in toto with a preformed biofilm of each strain (*S. epidermis, S. aureus, C. acnes* and *M. furfur*) showed a significative biofilm eradication ability. A known antibiofilm compound was selected for each strain as a positive control and the efficacy of the non-cross-linked HA hydrogel was tested in parallel. In Figure 2, the graphical representation of the antibiofilm activity is shown against a preformed biofilm for each strain. Since MTT was the chosen methodology, the results demonstrated the ability of the formulation to interfere with the metabolic activity of the biofilm, determined by the reduction of MTT according to the reference method (*n* = 2; *r* = 4) [19].

### 3.3. Evaluation of Cytotoxicity on Reconstructed Human Epidermis Infected 3D Model

Viability testing was performed in order to verify that the experimental model adopted remained in compliance with the acceptability criteria of the standard method (viability ≥ 50%). In order to evaluate the efficacy of our infected model, the cytotoxicity levels for each condition tested on Reconstructed Human Epidermis (RHE) inserts were evaluated. The results obtained showed a viability higher than 50% for each tested condition.

**Figure 3:**
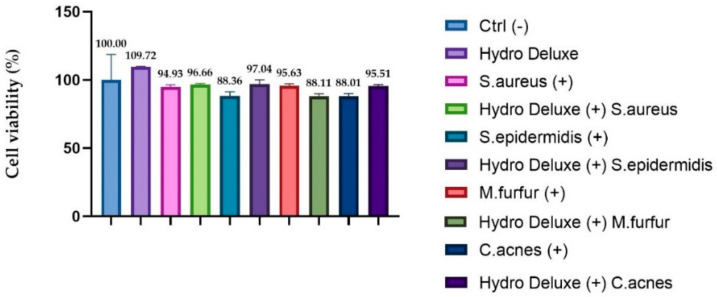
RHE inserts cell viability expressed as a percentage (%) compared to negative control Ctrl (-): RHE treated with DPBS; (+): RHE inserts treated with the selected strains (*S. aureus* ATCC 6538, *S. epidermidis* ATCC 12228, *C. acnes* ATCC 11827 and *M. furfur* ATCC 14521) Hydro Deluxe (+): RHE co-treated for 24 h with the HA-based formulation 100% (in toto) and the selected heat-killed strains; Hydro Deluxe: RHE inserts treated with for 24 h with the HA-based formulation 100% (in toto).

Figure 3 shows the viability of each insert after 24 h of treatment with the non-cross-linked HA hydrogel, with topical application of each heat-killed strain (*S. epidermis, S. aureus, C. acnes* and *M. furfur)* and with a co-treatment with hydrogel and each heat-killed strain. The percentage of viability was calculated with respect to the control (inserts treated only with Dulbecco Phosphate Buffer Saline solution (DPBS) (*n* = 1, replicates = 2).

### 3.4. Evaluation of Anti-Inflammatory Activity on Stimulated Reconstructed Human Epidermis

**Figure 4:**
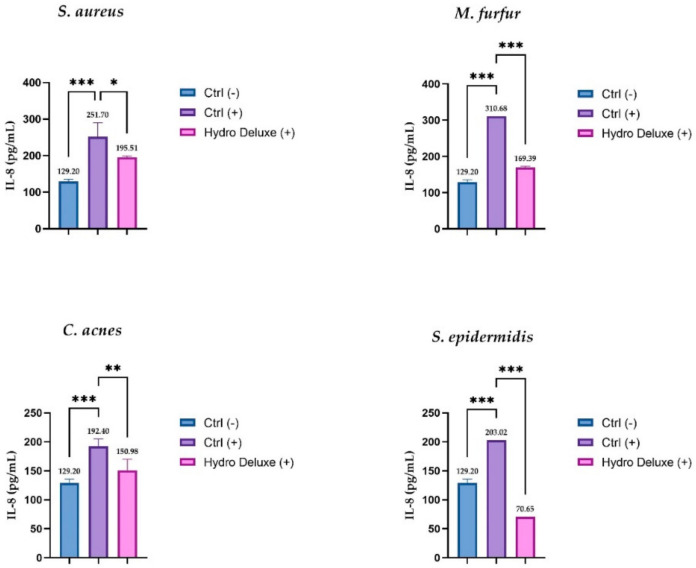
IL-8 amount (pg/mL) in medium after treatment with non-cross-linked HA hydrogel (Hydro Deluxe) in RHE inserts stimulated with the selected strains (*S. aureus* ATCC 6538, *S. epidermidis* ATCC 12228, *C. acnes* ATCC 11827 and *M. furfur* ATCC 14521). Ctrl (-): cells treated with DPBS; Ctrl (+): RHE stimulated with each of selected strains. Hydro Deluxe (+): RHE co-treated with selected strains and the non-cross-linked HA formulation 100% (*in toto*). Values of * *p* ≤ 0.05, ** *p* ≤ 0.01 and *** *p* ≤ 0.001 were considered statistically significant compared with respective controls by one-way ANOVA statistical analysis, followed by Bonferroni’s multiple comparisons as post-test.

The soothing effects of the HA-based formulation were evaluated, quantifying the expression of IL-8 by ELISA assay after topical application with the microbial strains (*S. epidermis, S. aureus, C. acnes* and *M. furfur*) in an RHE model. The IL-8 levels were expressed as concentration in pg/mL in RHE inserts infected with the microbial strains and treated with the hydrogel in toto compared to positive control (RHE inserts stimulated with the selected strains), as shown in Figure 4. The data demonstrated a remarkable effect on the release of IL-8, leading to a statistically significant reduction of the cytokine levels after 24 h of treatment and pro-inflammatory stimulation, compared to respective stimulated control (*n* = 2, replicates = 2).

## 4. Discussion

Keratinocytes are the primary type of cell that forms the epidermis, the outermost layer of the skin which plays the major role of interface between the host and the external environment [3]. Compared to the skin of the body, the scalp has peculiar features, due to a high follicular density and a high rate of sebum production by the activity of sebaceous glands (SGs). Sebum, in association with desquamated skin cells, can provide an enriched nutrient source for several microorganisms. Hyperkeratosis (scaling), pruritus, alopecia (hair loss) and inflammatory signs (erythema) are common symptoms of scalp disorders often related to a greater susceptibility of the scalp to bacterial or mycotic infections [28]. Scalp microbiome analyses showed that the most abundant bacteria found in the scalp are *C. acnes*, *S. aureus* and *S. epidermidis*; *M. furfur* is among the most common cutaneous commensal fungi of the scalp [29,30]. On undamaged skin, this resident microbiome provides a benefit to skin integrity and is considered non-pathological. However, under particular conditions which are still under study, the same strains can cause opportunistic infections. Factors such as excessive sebum production, water content, skin pH and personal hygiene are crucial in regulating skin microbiome proliferation and conditioning the scalp’s physiological microenvironment. Cutaneous microbiome dysbiosis is one of the trigger factors that can stimulate the production of immune modulators by keratinocytes and lead to the onset of inflammatory conditions related to many skin disorders. Among these, inflammatory states due to dandruff, atopic dermatitis (AD), seborrheic dermatitis (SD) and alopecia are among the most influenced by microbial over-colonization [7].

Nowadays, among the innovative treatments for scalp disorders—and in particular for the cicatricial and non-cicatricial forms of alopecia—mesotherapy or bio-revitalization is one of the most explored. The aim of hair mesotherapy is the restoration of the scalp physiology; this is accomplished by stimulating various biological responses via injecting active substances into scalp. There is no standardized formulation or definite protocol for the compounds and concentrations, but commonly-used substances in hair mesotherapy include minoxidil, finasteride, dutasteride, biotin, tretinoin, pantothenic acid, and other vitamins and minerals [20]. In our study, a non-cross-linked hyaluronic acid (HA) based formulation (Matex Lab S.p.a., Brindisi, Italy) containing 18 mg/mL of HA, Glycine, Proline and 0.01% CaHA, was investigated through a panel of in vitro tests in order to screen its potential efficacy for the treatment of scalp disorders. Since HA exhibits concentration-dependent antimicrobial activity, the first step was to verify that the hydrogel was effective against the four selected microbial strains (*C. acnes* ATCC 11827, *S. aureus* ATCC 6538, *S. epidermidis* ATCC 12228 and *M. furfur* ATCC 14521).

After 24 h of incubation of 10^6^ CFU/mL with 8 scalar dilutions in a ratio 1:2 (range tested 100–0.78%) at optimal growth conditions, the results showed that the growth rates of all selected microbial strains were sensitive with respect to the hydrogel formulation and that they decreased as the gradient concentration increased, demonstrating good antimicrobial activity. Together with the antimicrobial activity, we also investigated the ability of the formulation to reduce the metabolic activity of a preformed biofilm of the same strains. Biofilm aggregation between squamous cells or into the surface of sebaceous glands represents one of the most resistant forms of bacterial colonization that can lead to chronic inflammation. To better evaluate the antibiofilm activity of the formulation, bioactive compounds with this recognized ability were selected for each strain of the experimental panel. For *S. epidermidis*, Cinnamaldehyde trans-3-phenyl-2-propenal, the major compound found in cinnamon, was selected; for *S. aureus*, 2-Aminoimidazole (2-AI) was chosen; for *C. acnes*, 5,4′-trihydroxy-trans-stilbene Resveratrol was selected; and lastly, Ketaconazole was seleceted for the *M. furfur* preformed biofilm [21,22,23,24]. The results obtained showed a higher percentage of biofilm inhibition for each microbial strain; in particular, Hydro Deluxe showed an antibiofilm activity comparable to that exerted by 2-AI against *S. aureus* and was even more effective than the other positive controls selected against *S. epidermidis, M. furfur* and *P. acnes*. Data obtained showed higher antibiofilm activities for both bacteria and yeast, demonstrating a clinically important outcome that could lead to the development of better approaches toward chronic skin inflammations due to biofilm aggregations.

Since ethical considerations prevented direct inoculation of microorganisms onto human skin, and animal models do not represent an appropriate system for the different commensal microbial communities [31], an internal model based on Reconstructed Human Epidermis (RHE) inserts, stimulated with the selected heat-killed strains, was developed to screen the soothing effects of the formulation in a context closer to actual human skin under inflammatory conditions. RHE inserts were stimulated with a high concentration of microbial agents to mimic a context of dysbiosis in which the over-proliferation of bacteria plays a key role in the onset of the inflammatory state. Keratinocytes, including follicular keratinocytes and sebocytes, protect the host by producing different antimicrobial peptides (AMPs) of a broad-spectrum activity against bacteria, fungi, viruses, and parasites. Pattern recognition receptors on keratinocytes, such as Toll-like Receptors (TLRs), recognize pathogen-associated molecular patterns such as lipopolysaccharide (LPS), inducing the immune response and the secretion of AMPs, cytokines, and chemokines [29]. Among the chemokines produced by keratinocytes, with the main role of attracting neutrophils to the site of infection, interleukin (IL)-8 is one of the most expressed. Numerous scientific studies have shown that the strains selected for this study can stimulate its overexpression. In our overview, thermal inactivation was used as a method to preserve bacterial surface proteins able to initiate the inflammatory process by preventing the over-proliferation of bacterial cells. Several studies have described heat shock as a fixation method (together with inactivation by chemical agents, sonication, and UV irradiations) to inactivate bacterial cells and preserve their surface structures. LPS for gram-negative bacteria and lipopeptides for gram-positive bacteria are recognized as trigger factors capable of resisting thermal shock and activating the inflammatory cascade in keratinocytes by interacting with TLR4 and TLR2, respectively, with subsequent production of pro-inflammatory cytokines, such as IL-8 [26,32]. For a better evaluation of our in vitro model, the cytotoxicity of each condition was evaluated, and the results showed good viability, greater than 50% (threshold for RHE irritation test) for all conditions tested. Therefore, the high concentration of bacteria, the hydrogel formulation, and the synergy in the co-treatment with both, did not affect the viability of the keratinocytes. In order to evaluate the anti-inflammatory activity, IL-8 levels were analyzed in each condition and the results obtained confirmed a significant stimulation by the selected strains and a significant reduction of IL-8 levels induced by the tested formulation in the presence of the strains as pro-inflammatory stimulus. Regulation of the inflammatory events initiated or perpetuated by keratinocytes could represent an important strategy for therapeutic treatment of chronic inflammatory skin diseases.

Overall, the data collected demonstrated the anti-inflammatory, antimicrobial and antibiofilm activities of the tested hydrogel against the main pathogens responsible for the inflammatory states related to many scalp disorders, making the formulation a useful tool for mesotherapy practice. Subsequent investigations on the formulation’s potential ability to interfere with the production of sebum, or to exert a soothing action on dermal papilla cells, will be conducted in the near future, pending further in vivo investigations.

## Data Availability

Data are included in the text; raw data are available from the cor-responding author.
